# Digital Engagement and Predictors of Semaglutide Persistence and Weight Loss in Severe Obesity: 48-Week Observational Study

**DOI:** 10.2196/80119

**Published:** 2026-08-26

**Authors:** Sarah Tournayre, Jean-baptiste Bonnet, Jean Anitcheou, Marion Faivre, Catherine Boegner, Abdulkader Jalek, Dominique Jullien, Camille Le Rouzic, Vincent Attalin, Justine Myzia, Youadigue Kemba, Ariane Sultan, Antoine Avignon

**Affiliations:** 1 Nutrition-Diabetes Department, Montpellier University Hospital, Centre Hospitalier Universitaire de Montpellier, 191 Avenue Du Doyen Gaston Giraud, Montpellier, 34295, France, 33 467338402; 2 Joint Research Unit (UMR) 1302, Desbrest Institute of Epidemiology and Public Health, University of Montpellier, INSERM, Université de Montpellier, Montpellier, France; 3 PhyMedExp, INSERM U1046, National Centre for Scientific Research (CNRS) Joint Research Unit (UMR) 9214, University of Montpellier, Université de Montpellier, Montpellier, France

**Keywords:** severe obesity, semaglutide, digital engagement, treatment adherence, weight loss, behavioral predictors, GLP-1 receptor agonist, real-world study

## Abstract

**Background:**

Real-world persistence with antiobesity pharmacotherapy is suboptimal: only 32% to 50% of patients persist with glucagon-like peptide-1 receptor agonists at 12 months, and fewer than 15% reach the 2.4 mg/week target dose. Digital platforms may support engagement, but their role remains poorly characterized.

**Objective:**

This study aimed to evaluate whether baseline engagement with a structured digital assessment platform (Aviitam) predicts 48-week persistence and mediates weight-loss outcomes in adults with severe obesity initiating semaglutide 2.4 mg/week and to identify clinical and behavioral predictors of pharmacological response.

**Methods:**

In this prospective 48-week single-center observational cohort, all consecutive adults (≥18 y) with a BMI ≥40 kg/m² initiating semaglutide 2.4 mg/week through the French early-access program were enrolled. Baseline engagement with the Aviitam digital assessment platform was operationalized as a 3-level ordinal variable (never used, partial use, and full completion of validated questionnaires), reflecting the patient’s responses to a nonenforced preclinical request. The primary outcome was 48-week persistence; secondary outcomes were full-dose attainment, percentage weight change, and dose-response. Adjusted logistic and linear regressions estimated the engagement-persistence and engagement-weight-loss associations. A Baron-Kenny mediation analysis with bootstrap CIs decomposed the engagement-weight-loss association. Missing 48-week weights were imputed using multiple imputation (m=20) as a sensitivity analysis. Among persistent participants who completed all 7 baseline questionnaires (n=91), behavioral-scale associations with weight change were analyzed using Benjamini-Hochberg false-discovery-rate correction.

**Results:**

Of 191 participants (mean age 51.7, SD 6.8 y; 61/191, 31.9% male; mean BMI 45.7, SD 6.8 kg/m²), 37 (19.4%) had never used the platform, 32 (16.8%) used it partially, and 122 (63.9%) fully completed the questionnaires. At 48 weeks, 142 (74.3%) remained on semaglutide, and 124 (87.3%) of them reached the 2.4 mg/week dose. Persistence followed a monotonic gradient across engagement levels (22/37, 59.5%; 23/32, 71.9%; 97/122, 79.5%; *P*=.02), and each engagement increment was associated with higher odds of persistence (adjusted odds ratio 1.62, 95% CI 1.05‐2.50; *P*=.03), unchanged under multiple imputation. Mean weight loss was 11.8% (SD 8.4%) in the full cohort and 14.6% (SD 7.3%) among persistent participants, with a steep dose-response (mean 3.9%, SD 5.8% in discontinuers to mean 15.3%, SD 7.2% at 2.4 mg/week; *P*<.001). Mediation analysis showed 48-week persistence mediated approximately 69% of the engagement–weight-loss association; the direct effect was nonsignificant. The Intuitive Eating Scale-2 was the only behavioral predictor surviving false-discovery-rate correction (*q*=0.05).

**Conclusions:**

Baseline engagement with a structured digital assessment platform predicts 48-week semaglutide persistence in a graded manner; low-engagement patients can be identified at initiation and supported with targeted strategies to maintain persistence and achieve full-dose titration.

## Introduction

In the context of rising obesity prevalence [1] and persistent challenges in long-term weight management [2], digital health solutions are increasingly positioned as critical components of chronic disease care [3]. Once-weekly semaglutide 2.4 mg has redefined pharmacological obesity treatment, producing 14% to 17% mean weight loss in phase 3 trials [4,5]. Translating this efficacy into real-world effectiveness is challenging: administrative datasets show that only 32% to 50% of patients persist with glucagon-like peptide-1 receptor agonists (GLP-1 RAs) at 12 months, and fewer than 15% reach the 2.4 mg/week target dose [6-10]. The mechanisms behind this effectiveness gap, particularly whether the rate-limiting step is persistence, dose attainment, or heterogeneity of biological response, remain underexplored, and populations with severe obesity (BMI ≥40 kg/m²) are typically underrepresented in trials despite carrying the largest disease burden.

Digital tools allow systematic, low-burden capture of behavioral and psychosocial determinants of obesity that are otherwise difficult to collect at scale, and they can structure multidisciplinary care around patient-level data [3,11]. In obesity care specifically, several digital platforms have been evaluated as adjuncts to lifestyle interventions (eg, app-based behavioral programs and commercial digital weight-management services), and a small number have been studied alongside pharmacotherapy. However, most published evidence concerns digital interventions as therapeutic agents rather than digital assessment as a marker of patient phenotype, and few studies have evaluated whether a preclinical engagement signal predicts subsequent treatment persistence. This distinction matters clinically: if engagement acts as a marker of patient characteristics, low-engagement patients can be identified at initiation and supported with intensified follow-up; if engagement acts as a therapeutic intervention, the educational content itself should be optimized. A joint advisory by the American College of Lifestyle Medicine, the American Society for Nutrition, the Obesity Medicine Association, and the Obesity Society has called for digital tools to enhance accessibility, self-monitoring, and engagement with nutritional and lifestyle interventions during GLP-1 therapy [11], but the empirical basis remains thin.

We hypothesized that the patient’s response to a nonenforced preclinical request to complete a structured digital assessment pathway plausibly reflects a composite of motivation, prioritization, time availability, digital and health literacy, and trust in the care process, and that this composite is associated with higher 48-week persistence and, through persistence, with greater weight loss. Persistence is defined here as the patient still receiving semaglutide at any dose at the planned 48-week visit. The present study does not attempt to disentangle the components of this composite, nor does it test whether the platform passively marks them or actively contributes to persistence.

In a 48-week prospective observational cohort of adults with severe obesity initiating semaglutide 2.4 mg/week through the French early-access program, we addressed 3 questions in order of priority. First, does baseline engagement with the Aviitam digital assessment platform, operationalized in a graded, ordinal manner, predict 48-week persistence? Second, does it predict percentage weight change at 48 weeks, and what is the dose-response gradient with the maintained semaglutide dose? Third, among persistent participants who completed all 7 validated baseline questionnaires (n=91), which clinical and behavioral characteristics are independently associated with the magnitude of weight loss, with multiplicity controlled by the Benjamini-Hochberg false-discovery-rate (FDR) correction? A descriptive mediation decomposition of the engagement-weight-loss association is reported for completeness.

## Methods

### Study Design and Setting

This was a prospective observational cohort study conducted in the Nutrition-Diabetes Department of Montpellier University Hospital (France) between April 2022 and April 2023. Reporting follows the STROBE (Strengthening the Reporting of Observational Studies in Epidemiology) statement (Checklist 1) [12]. Follow-up was conducted over 48 weeks, with scheduled visits at baseline and at weeks 12, 24, and 48. The 48-week follow-up window was chosen because it aligns with the primary end point of the step 1 trial [4], encompasses the full dose-titration phase of semaglutide and the early plateau of weight loss, matches the routine clinical pathway in our department, and corresponds to the duration of structured monitoring guaranteed by the French early-access program.

### Aviitam Digital Assessment Platform and Engagement Variable

Aviitam is a secure digital platform deployed in French clinical centers since 2014, supporting more than 30,000 users in the longitudinal management of obesity and other chronic conditions. In routine departmental practice, patients are enrolled on the platform at the time of appointment scheduling and assigned a standardized obesity care pathway to complete from home before their first clinical visit. At the time of scheduling, the departmental secretariat informs patients verbally that completion of the pathway is requested before the first visit; the platform does not technically enforce completion, and the appointment-request button can be activated whether or not the questionnaires have been filled in. The pathway includes self-reported medical history, lifestyle, nutrition, sleep, mental health, and quality-of-life items, together with 7 validated patient-reported scales: the Intuitive Eating Scale-2 (IES-2) [13], the Binge Eating Scale [14], the Hospital Anxiety and Depression Scale [15], the Perceived Stress Scale [16], the Epworth Sleepiness Scale (ESS) [17], the Insomnia Severity Index [18], and the Ricci-Gagnon Physical Activity Questionnaire [19]. Aviitam also offers optional health-education content (short videos, written modules, and behavioral exercises on nutrition, physical activity, sleep hygiene, and stress management); this content is not part of the assigned pathway, patients are not actively directed to it, and its use is neither prescribed nor monitored. Pathway completion therefore reflects the patient’s behavioral response to a nonenforced preclinical instruction.

Baseline engagement was operationalized from using this preclinical pathway-completion behavior and categorized into three ordinal levels: (0) never used the platform, (1) used the platform but did not complete all validated questionnaires, and (2) completed all validated questionnaires. Because the pathway functions as a self-administered assessment tool rather than a therapeutic intervention, and clinical follow-up was identical across engagement levels, this measure captures the patient’s behavioral response to the preclinical instruction rather than their exposure to a digital intervention. The composite of factors that this behavior likely reflects (motivation, prioritization, time availability, digital and health literacy, language proficiency, and trust in the care process) is discussed in the “Limitation” section. The ordinal variable was used as the primary engagement exposure; a binary variable (full completion vs not) was analyzed in a sensitivity analysis.

### Eligibility Criteria

Inclusion criteria were adults (≥18 y) initiating semaglutide 2.4 mg/week during the study window through the French early-access program (Accès Précoce), with BMI ≥40 kg/m² and at least 1 obesity-related complication (hypertension, dyslipidemia, established cardiovascular disease, or treated obstructive sleep apnea [OSA]). The BMI threshold of ≥40 kg/m² reflected the eligibility rules of the early-access program and was retained for our analysis to focus on severe obesity, the population least represented in pivotal trials. Exclusion criteria were prior GLP-1 RA exposure at therapeutic doses (>0.25 mg/week, mainly in patients with type 2 diabetes [T2D]), to capture true treatment initiation; monogenic, syndromic, or secondary obesity, because the biological response to GLP-1 pathway activation may differ in these conditions; pregnancy or planned bariatric surgery during follow-up, to avoid confounding of the weight-loss outcome; active cancer; and an estimated glomerular filtration rate <30 mL/min/1.73 m², in line with the label safety profile of semaglutide.

### Sampling and Recruitment

**Figure 1.**
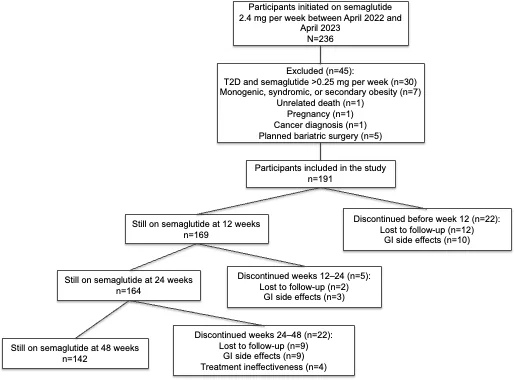
Participant flow diagram. Out of 236 patients initiating semaglutide 2.4 mg/week at the Montpellier Nutrition-Diabetes Department between April 2022 and April 2023, 45 were excluded for protocol-defined reasons (prior glucagon-like peptide-1 receptor agonist exposure, n=30; syndromic or secondary obesity, n=7; planned bariatric surgery, n=5; active cancer, n=1; pregnancy, n=1; and unrelated death, n=1), yielding an analytic cohort of 191 participants. GI: gastrointestinal intolerance; T2D: type 2 diabetes.

The sampling frame comprised all adults consecutively initiating semaglutide 2.4 mg/week through the French early-access program at the Montpellier Nutrition-Diabetes Department between April 2022 and April 2023. Sampling was exhaustive: all eligible patients during the recruitment window were enrolled, and no patient declined participation. No formal a priori sample-size calculation was performed because the recruitment window was constrained by the duration of the early-access program. Post hoc, with 191 participants and the observed (n=49) 25.7% discontinuation rate, the study had approximately 80% power at an *α* level of .05 (2-sided) to detect a per-level odds ratio (OR) of 1.79 (or 0.56 in the protective direction) for the ordinal engagement-persistence association, exceeding the magnitude of the adjusted effect we observed. Sampling outcomes are detailed in the participant flow diagram (Figure 1): of 236 patients initiating semaglutide 2.4 mg/week during the recruitment window, 45 were excluded for protocol-defined reasons (prior GLP-1 RA exposure, n=30; syndromic or secondary obesity, n=7; planned bariatric surgery, n=5; active cancer, n=1; pregnancy, n=1; unrelated death, n=1), yielding the final analytic cohort of 191 participants.

### Treatment Protocol

Semaglutide was initiated at 0.25 mg/week and titrated every 4 weeks up to 2.4 mg/week, based on tolerability. While longer-term use was possible, analyses were restricted to the planned 48-week follow-up. The French early-access program provided semaglutide at no cost to patients prior to marketing authorization for obesity, thereby removing financial barriers and ensuring structured clinical follow-up. Continuation and dose escalation were not externally incentivized or protocol-mandated; clinicians made titration decisions based on tolerability, reflecting routine practice. The semaglutide dose maintained at 48 weeks was defined as the dose recorded in the patient’s electronic prescription at the 48-week visit; discontinuation was operationalized as the absence of an active semaglutide prescription at 48 weeks.

### Data Collection and Measurements

Anthropometric measurements (height, weight, and waist circumference) and blood pressure were recorded at each visit. Laboratory parameters (HbA_1c_, creatinine, lipid profile, and C-reactive protein) were measured at certified local laboratories; the estimated glomerular filtration rate was calculated using the Chronic Kidney Disease Epidemiology Collaboration (CKD-EPI) equation. Comorbidities, family history of T2D and obesity, weight history (highest and lowest reported adult weight), and lifestyle factors were documented during structured clinical interviews. Socioprofessional category (CSP), based on the French national classification, was recorded as a standardized proxy for socioeconomic position and was grouped into: (1) executives and self-employed, (2) employees, and (3) other (retirees, students, homemakers, and unemployed individuals). The heterogeneity of the third category was addressed by a sensitivity analysis that coded retirees separately (Table S1 in Multimedia Appendix 1).

### Outcomes

The primary outcome was treatment persistence at 48 weeks, defined as the patient still receiving semaglutide at any dose. Secondary outcomes were: (1) attainment of the 2.4 mg/week target dose among persistent participants; (2) percent body weight change at weeks 12, 24, and 48; (3) the dose-response relationship between the dose maintained at 48 weeks and percent weight change; and (4) clinical, metabolic, and behavioral predictors of weight loss.

### Statistical Analysis

Descriptive statistics are reported as mean (SD) for continuous variables and n (%) for categorical variables; medians and IQR are used where the distribution is markedly nonnormal. Baseline characteristics were compared between engagement levels using Student 2‑tailed *t* tests, Wilcoxon rank-sum tests, Cochran-Armitage trend tests, or chi-square tests as appropriate. Logistic regression identified factors associated with 48-week persistence, with covariates entered if associated at *P*≤.10 in univariate analysis, together with the a priori confounders age, sex, and baseline BMI. The ordinal engagement variable was entered into the model as a continuous trend predictor; a binary specification (full completion vs not) was tested in sensitivity analyses. Linear regression evaluated percentage weight change. The complete statistical analysis plan is provided in Multimedia Appendix 2, and the assumed causal structure of these analyses, including measured and unmeasured confounding, is illustrated as a directed acyclic graph in Multimedia Appendix 3.

A formal mediation analysis decomposed the effect of baseline engagement on percent weight change at 48 weeks into a direct effect (c’) and an indirect effect mediated by 48-week persistence (a×b), following the Baron-Kenny framework [20], with bootstrapped 95% CIs for the indirect effect (1000 resamples) [21]. The analysis was adjusted for age, sex, and baseline BMI and run with both binary and ordinal engagement exposures.

Missing 48-week weight outcomes (n=49, 25.7%) were concentrated among patients who discontinued semaglutide and followed a monotone attrition pattern. The primary analysis used last-observation-carried-forward imputation, consistent with the routine clinical convention in our department. Multiple imputation by chained equations was performed as a sensitivity analysis (m=20, a Bayesian Ridge per-variable estimator drawing from the posterior, the IterativeImputer class from *scikit‑learn* version 1.7 [22-24]) with age, sex, baseline BMI, baseline weight, ordinal engagement, persistence, full-dose attainment, treated OSA, family history of T2D, baseline T2D, historical lowest reported adult weight, and baseline glycated hemoglobin (HbA_1c_) as auxiliary variables; estimates were pooled using Rubin’s rules. The complete-case analysis is reported for transparency, although it is not informative for the mediation analysis because removing patients with missing 48-week weight eliminates all discontinued patients and, therefore, the variability of the persistence variable. The side-by-side comparison of estimates under the 3 strategies is presented in the “Results” section.

An exploratory subgroup analysis among persistent participants with fully completed validated baseline questionnaires (n=91) tested associations between the 7 behavioral scales and percent weight change. We restricted this analysis to participants with complete baseline scales because partial responders had insufficient items to compute validated questionnaire totals; a sensitivity analysis using item-level multiple imputation on partial responders is reported in Table S2 in Multimedia Appendix 1. Multiplicity was controlled using the Benjamini-Hochberg false discovery rate (FDR) procedure [25]. A multivariable linear model included behavioral predictors with raw *P*<.10 together with the a priori clinical confounders age, sex, and baseline BMI.

Sensitivity analyses stratified the outcomes by baseline T2D status. All analyses used R version 4.3.3 (R Foundation for Statistical Computing) and Python 3.10 (Python Software Foundation) for the mediation bootstrap and multiple imputation procedures. Two-sided *P*<.05 was considered statistically significant for the primary hypotheses, with FDR-corrected *q*<.05 for the exploratory behavioral analyses.

### Ethical Considerations

The study was approved by the Montpellier Ethics Committee (Comité Scientifique et Éthique de Montpellier, approval number 2024-05-061) and classified as a noninterventional observational study (RIPH3) under French law. Written informed consent for the use of anonymized data was obtained from all participants at the baseline clinical visit. Data were anonymized at the source; access to identifiable data was restricted to the treating clinicians, and analysis datasets contained only pseudonymized identifiers. The study complied with French data-protection regulations and the European General Data Protection Regulation. No participant compensation was provided at any stage.

## Results

### Participant Flow and Cohort Description

Between April 2022 and April 2023, 236 patients initiated semaglutide 2.4 mg/week at the Montpellier Nutrition-Diabetes Department. After exclusions (prior GLP-1 RA exposure, n=30; syndromic or secondary obesity, n=7; planned bariatric surgery, n=5; active cancer, n=1; pregnancy, n=1; and unrelated death, n=1), 191 participants were included (Figure 1). Mean age was 51.7 (SD 13.6) years; 31.9% were male; mean BMI was 45.7 (SD 6.8) kg/m². Baseline T2D was present in 11.0% (21/191), family history of T2D in 29.8% (57/191), treated OSA in 55.0% (105/191), and prior bariatric surgery in 25.7% (49/191). Baseline characteristics for the full cohort, and stratified by engagement level, are reported in Table 1.

**Table 1.** Baseline characteristics of the cohort overall and stratified by baseline engagement level.

Characteristics	Total (N=191)	Never used (n=37)	Partial use (n=32)	Full completion (n=122)
Demographics				
Age (y), mean (SD)	51.7 (13.6)	52.8 (14.4)	51.1 (12.8)	51.4 (13.6)
Male, n (%)	61 (31.9)	14 (37.8)	10 (31.3)	37 (30.3)
Anthropometrics, mean (SD)				
Body weight (kg)	126.5 (24.5)	124.5 (25.8)	125.7 (22.7)	127.4 (24.6)
BMI (kg/m²)	45.7 (6.8)	44.8 (6.2)	46.1 (7.1)	45.9 (7.0)
Waist circumference (cm)	131.1 (14.1)	130.9 (14.6)	131.6 (14.9)	131.0 (13.9)
Lowest reported adult weight (kg)	76.6 (20.3)	76.3 (17.9)	75.3 (21.3)	77.0 (20.8)
Highest reported adult weight (kg)	134.9 (28.8)	133.1 (34.0)	136.6 (26.7)	135.0 (27.5)
Clinical characteristics, n (%)				
Hypertension	98 (51.3)	17 (45.9)	13 (40.6)	68 (55.7)
Dyslipidemia	67 (35.1)	10 (27.0)	11 (34.4)	46 (37.7)
Established cardiovascular disease	29 (15.2)	6 (16.2)	5 (15.6)	18 (14.8)
Treated obstructive sleep apnea	105 (55.0)	25 (67.6)	18 (56.3)	62 (50.8)
Type 2 diabetes	21 (11.0)	4 (10.8)	3 (9.4)	14 (11.5)
Family history of type 2 diabetes	57 (29.8)	11 (29.7)	9 (28.1)	37 (30.3)
Family history of obesity	105 (55.0)	19 (51.4)	19 (59.4)	67 (54.9)
Prior bariatric surgery	49 (25.7)	14 (37.8)	8 (25.0)	27 (22.1)
Socioprofessional category^a^, n (%)				
Executives or self-employed	65 (34.0)	9 (24.3)	7 (21.9)	49 (40.2)
Employees	54 (28.3)	9 (24.3)	10 (31.3)	35 (28.7)
Other (retirees, students, homemakers, and unemployed)	72 (37.7)	19 (51.4)	15 (46.9)	38 (31.1)
Biochemistry, mean (SD)				
HbA_1c_^b^ (%)	5.9 (0.6)	5.9 (0.6)	5.9 (0.6)	5.8 (0.6)
eGFR^c^ CKD-EPI^d^ (mL/min/1.73 m²)	90.5 (18.2)	92.8 (14.9)	91.9 (16.4)	89.3 (19.7)

a *χ*² *P*=.02 for socioprofessional category distribution across engagement levels; all other between-level comparisons had *P*>.10.

b HbA_1c_: glycated hemoglobin.

c eGFR: estimated glomerular filtration rate.

d CKD-EPI: Chronic Kidney Disease Epidemiology Collaboration.

At baseline, 37 (19.4%) participants had never used the Aviitam platform, 32 (16.8%) used it partially, and 122 (63.9%) completed all validated questionnaires. Engagement levels were comparable across demographic, anthropometric, and clinical characteristics, with the exception of CSP distribution (*χ*²; *P*=.02); executives and self-employed participants were overrepresented among fully engaged patients. The engagement-persistence and engagement-weight-loss associations reported below were adjusted for CSP.

### Baseline Engagement and 48-Week Persistence

**Figure 2.**
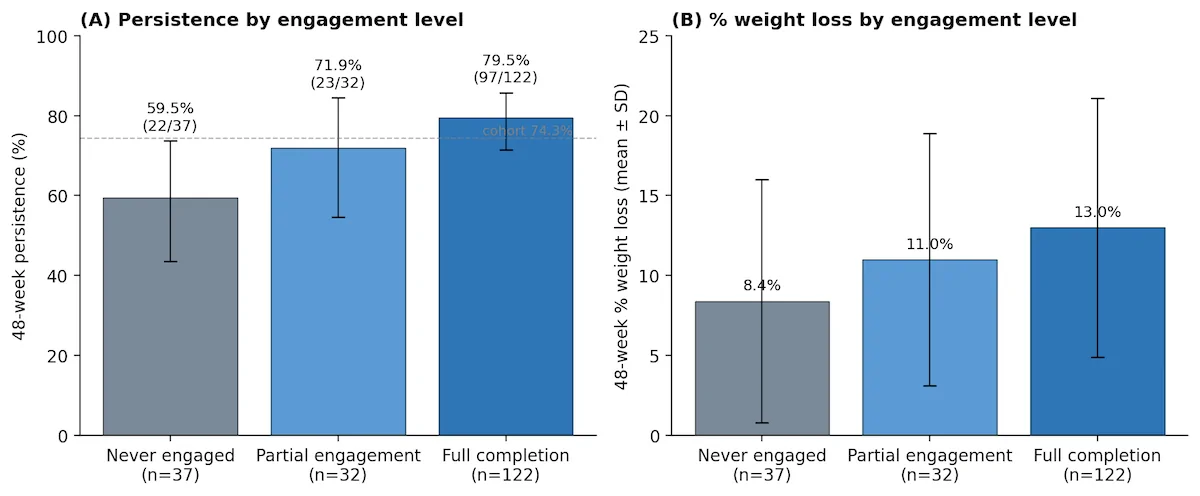
Baseline engagement gradient and clinical outcomes. (A) Proportion of participants persisting with semaglutide at 48 weeks across the 3 engagement levels (never used the platform, partial use, and full completion of validated questionnaires). (B) Mean percentage weight loss at 48 weeks by engagement level; error bars indicate SDs. The adjusted odds ratio per ordinal increase in engagement level for 48-week persistence was 1.62 (95% CI 1.05‐2.50; *P*=.03) from multivariable logistic regression adjusted for age, sex, baseline BMI, socioprofessional category, and historical lowest reported adult weight.

Persistence rates showed a monotonic gradient across engagement levels: 59.5% (22/37) among never-users, 71.9% (23/32) among partial users, and 79.5% (97/122) among full completers (*χ*²; *P*=.015; Figure 2A). In multivariable logistic regression adjusted for age, sex, baseline BMI, CSP, and historical lowest reported adult weight, each increment in ordinal engagement level was associated with higher odds of 48-week persistence (adjusted OR 1.62, 95% CI 1.05‐2.50; *P*=.03). The association was unchanged in the multiple-imputation sensitivity analysis (adjusted OR 1.62, 95% CI 1.05‐2.50; *P*=.03). When engagement was specified as a binary variable (full completion vs not), the adjusted OR for persistence was 2.28 (95% CI 1.11‐4.55; *P*=.03). The historical lowest reported adult weight was independently associated with persistence (OR 0.97, 95% CI 0.95‐0.99 per kg; *P*=.01); no other clinical variable reached significance. Model discrimination was modest (C-statistic 0.67).

### Persistence, Full-Dose Attainment, and Dose-Response in Weight Loss

Among the 142 persistent participants, 124 (87.3%) reached the 2.4 mg/week target dose. Documented reasons for early discontinuation among the 49 nonpersistent participants were gastrointestinal intolerance (n=21), loss to follow-up (n=24), and perceived ineffectiveness (n=4). Among the persistent participants, 124 (87.3%) reached the 2.4 mg/week target dose.

**Figure 3.**
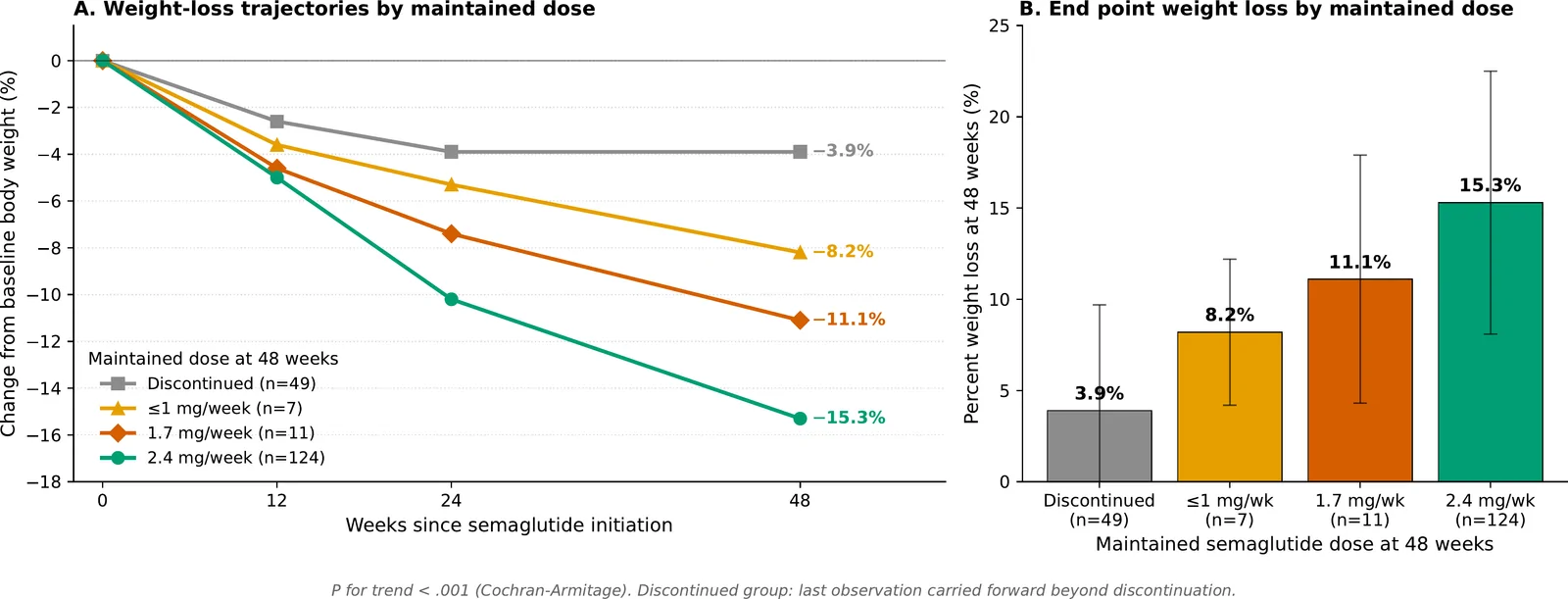
Pharmacological dose-response in real-world weight loss. (A) Weight-loss trajectories over 48 weeks in the full cohort (n=191), stratified by the semaglutide dose maintained at 48 weeks. Lines show group means; markers indicate measurements at weeks 0, 12, 24, and 48. For patients who discontinued, the last observed weight was carried forward beyond the time of discontinuation. (B) End point percentage weight loss at 48 weeks by maintained-dose category; error bars show SDs. *P*<.001 (Cochran-Armitage). GLP-1 RA: glucagon-like peptide-1 receptor agonist.

Weight loss at 48 weeks was strongly graded by the maintained dose (Figure 3). Mean percentage weight change was 3.9% (SD 5.8%) in patients who discontinued (n=49), 8.2% (SD 4.0%) at ≤1 mg/week (n=7), 11.1% (SD 6.8%) at 1.7 mg/week (n=11), and 15.3% (SD 7.2%) at 2.4 mg/week (n=124) (*P*<.001, Cochran-Armitage). In the full cohort, mean weight loss reached 4.3% (SD 3.5%) at 12 weeks, 8.2% (SD 5.8%) at 24 weeks, and 11.8% (SD 8.4%) at 48 weeks. Among persistent participants, the corresponding values were 4.9% (SD 3.2%), 9.7% (SD 5.0%), and 14.6% (SD 7.3%). At 48 weeks, 76.4% (146/191) of the full cohort lost ≥5% of baseline weight, 58.1% (111/191) lost ≥10%, and 35.6% (68/191) lost ≥15%. Among persistent participants, 92.3% (131/142) lost ≥5%, 74.7% (106/142) lost ≥10%, and 45.1% (64/142) lost ≥15%. In a multivariable analysis among persistent participants, adjusted for age, sex, baseline BMI, treated OSA, family history of T2D, and historically lowest reported adult weight, reaching the 2.4 mg/week target dose was the strongest clinical predictor of weight loss (β=+4.8 percentage points; 95% CI 1.4-8.2; *P*=.006).

### Mediation of the Engagement−Weight-Loss Association by Persistence

**Figure 4.**
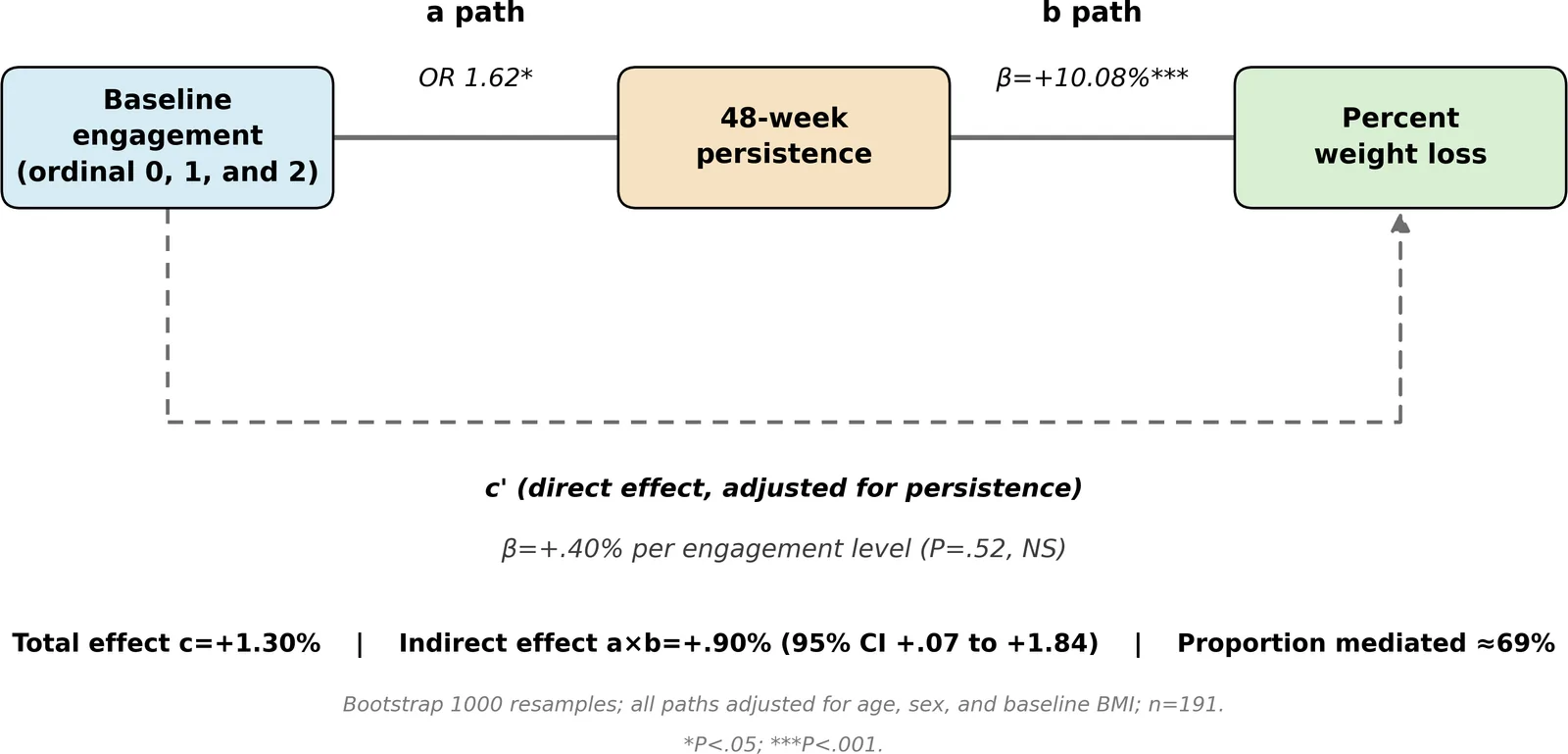
Mediation analysis of the effect of baseline engagement on percentage weight change at 48 weeks. The a-path is the effect of engagement on 48-week persistence; the b-path is the effect of persistence on weight loss; the C’-path is the residual direct effect of engagement on weight loss after adjustment for persistence. The a×b indirect effect was estimated by bootstrapping (1000 resamples): +0.90 percentage points per engagement level (95% CI 0.07‐1.84), accounting for approximately 69% of the total effect. All models were adjusted for age, sex, and baseline BMI. OR: odds ratio.

The decomposition was based on the assumed causal structure illustrated in Multimedia Appendix 3. In the formal mediation analysis (Figure 4), the total effect of ordinal engagement on percentage weight change at 48 weeks was +1.30 percentage points per level (95% CI −0.14 to +2.74; *P*=.08, adjusted for age, sex, and baseline BMI). After additional adjustment for 48-week persistence, the direct effect of engagement was attenuated to +0.40 percentage points per level (95% CI −0.83 to +1.63; *P*=.52), while 48-week persistence itself was strongly associated with greater weight loss (β=+10.1 percentage points; *P*<.001). The bootstrap estimate of the indirect effect was +0.90 percentage points per engagement level (95% CI 0.07-1.84), representing approximately 69% of the total effect. The same qualitative pattern was observed with binary completion as the exposure (total effect +2.56%; *P*=.03; direct effect +1.18%; *P*=.25; indirect effect +1.38%, 95% CI 0.15-2.85). In the multiple-imputation sensitivity analysis, the direct effect remained nonsignificant (β=+.11 percentage points per level; *P*=.90) and the indirect-effect pattern was preserved (see Table 2 for a side-by-side comparison).

Sensitivity to the missing-weight handling strategy is summarized in Table 2.

**Table 2.** Engagement-persistence and mediation parameters under 3 strategies for handling missing 48-week weight outcomes.

Parameter	LOCF^a^ (primary	Complete case	Multiple imputation^b^
Outcome model, n	191	142	191
Engagement → persistence, adjusted OR^c^ per ordinal level (95% CI; *P* value)	1.62 (1.05‐2.50; .03)	Not estimable^d^	1.62 (1.05‐2.50; .03)
Total effect on % weight change, β per level (*P* value)	+1.30 (.08)	−0.16 (.85)	+0.30 (.74)
Direct effect after adjustment for persistence β (*P* value)	+0.40 (.52)	−0.16 (.85)	+0.11 (.90)
Indirect effect via persistence β (% mediated)	+0.90 (~69)	Not estimable^d^	+0.19 (~63)

a LOCF: last observation carried forward.

b MI: multiple imputation.

c OR: odds ratio.

d Complete-case analysis removes all 49 discontinued patients (all of whom had missing 48-week weight after undoing LOCF), eliminating variability in the persistence variable. The a-path and persistence-mediated effect are therefore not informative under complete cases. Multiple imputation was performed using IterativeImputer (scikit-learn) with a Bayesian Ridge per-variable estimator and m=20 imputations; estimates pooled with Rubin's rules.

### Clinical Predictors of Weight Loss Among Persistent Participants

In the multivariable model among persistent participants (n=142), full-dose attainment (β=+4.8 percentage points, 95% CI 1.4-8.2; *P*=.006), absence of treated OSA (β=+3.2 percentage points, 95% CI 1.0-5.5; *P*=.006), and lower historical lowest reported adult weight (β=−0.09 percentage points per kg higher, 95% CI −0.16 to −0.01; *P*=.018) were each independently associated with greater percentage weight loss. Family history of T2D showed a nonsignificant trend toward smaller weight loss (β=−2.0 percentage points; *P*=.11). Current T2D status was not independently associated with weight loss in this cohort.

### Exploratory Behavioral Subgroup Analysis With FDR Correction

**Figure 5.**
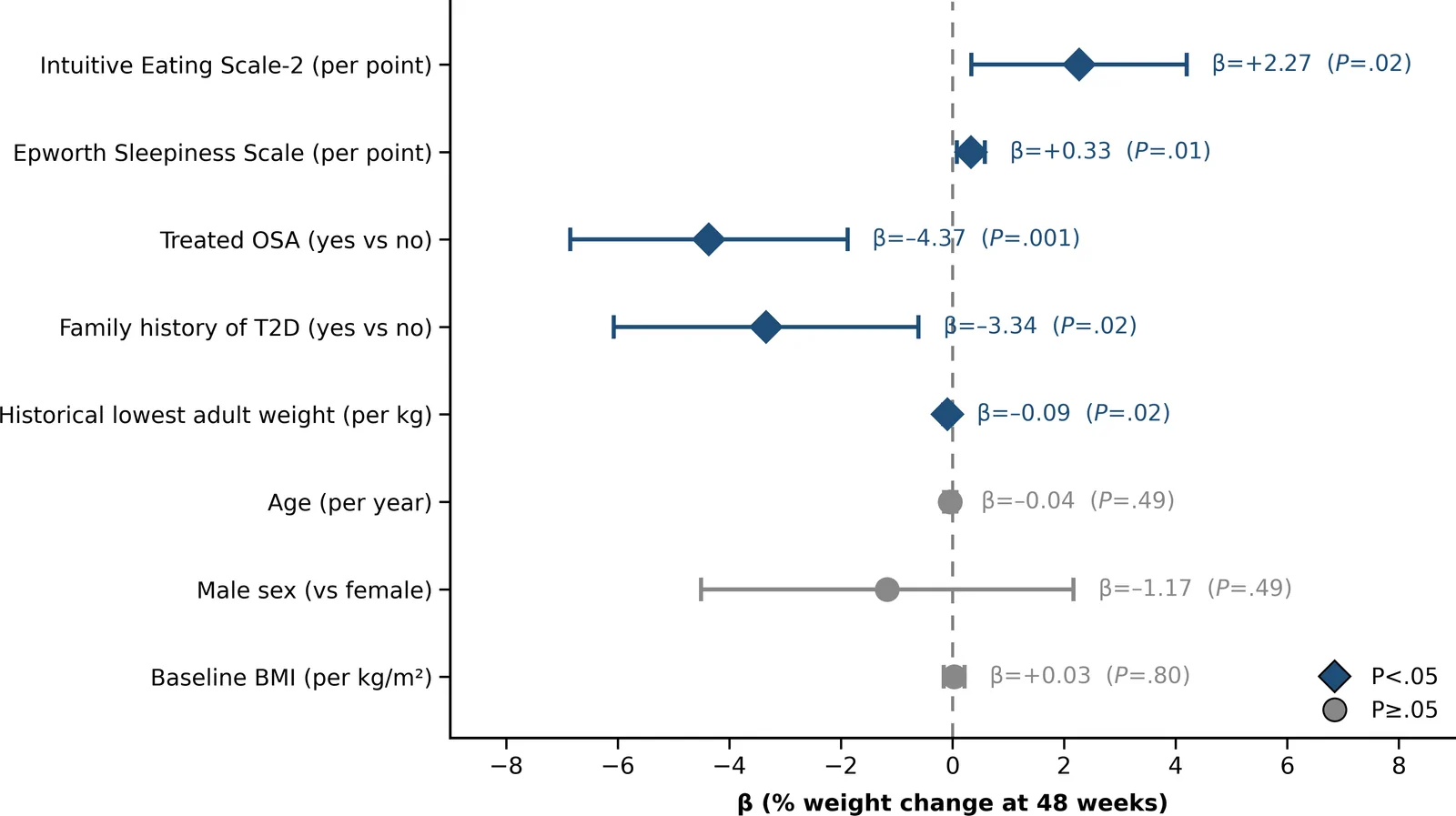
Forest plot of the multivariable linear regression of 48-week percent weight change on baseline behavioral and clinical predictors in the behavioral subgroup (n=91). Each point shows the regression coefficient (β) with its 95% CI. The vertical reference line marks β=0 (no effect). Negative values indicate smaller weight loss; positive values indicate greater weight loss. The Intuitive Eating Scale-2 association survived Benjamini-Hochberg false-discovery-rate correction across the 7 behavioral scales in univariate testing. OSA: obstructive sleep apnea; T2D: type 2 diabetes.

Baseline characteristics of the 91 behavioral subgroup are reported in Table S in Multimedia Appendix 1. Univariate associations between the 7 behavioral scales and percent weight change were tested with Benjamini-Hochberg FDR correction (Table S4 in Multimedia Appendix 1). IES-2 showed the strongest association (β=+2.89 percentage points per point; raw *P*=.007; *q*=.05), approaching the multiplicity-corrected significance threshold. The ESS was nominally associated (raw *P*=.03) but did not survive FDR correction (*q*=.09). The remaining 5 scales showed no association (all raw *P*>.10; all *q*>.20). In a multivariable model adjusted for age, sex, and baseline BMI (Table S5 in Multimedia Appendix 1; forest plot in Figure 5), IES-2 (β=+2.27 percentage points per point; *P*=.02), ESS (β=+.33 percentage points per point; *P*=.009), treated OSA (β=−4.37 percentage points; *P*=.001), family history of T2D (β=−3.34 percentage points; *P*=.02), and historical lowest reported adult weight (β=−.09 percentage points per kg; *P*=.02) were each independently associated with percent weight change. Because only the IES-2 association survived FDR correction, all behavioral findings beyond IES-2 are presented as hypothesis-generating.

### Sensitivity Analyses by T2D Status

Among the 21 participants with baseline T2D, 48-week persistence was 76.2% (16/21) and mean percentage weight loss was 8.9% (SD 7.4%), compared with 74.1% (126/170) persistence and 12.2% (SD 8.4%) weight loss in the 170 participants without T2D (*P*=.07; Table S6 in Multimedia Appendix 1). The direction and statistical significance of all primary predictor effects, including the engagement-persistence and mediation results, were unchanged when stratified by baseline T2D status.

## Discussion

### Principal Findings

In this prospective 48-week observational study of 191 adults with severe obesity initiating semaglutide 2.4 mg/week through the French early-access program, 3 findings emerged. First, baseline engagement with the Aviitam digital assessment platform predicted 48-week persistence in a graded manner across 3 ordinal levels (persistence was 22/37, 59.5%; 23/32, 71.9%; 97/122, 79.5%) in nonengagers, partial engagers, and full completers, respectively; the adjusted OR per level was 1.62, 95% CI 1.05-2.50). A descriptive decomposition of the engagement–weight-loss association attributed approximately 69% of that association to 48-week persistence, a pattern equally consistent with a passive-marker interpretation and an active-aid interpretation. Second, real-world effectiveness in a structured multidisciplinary setting approached the efficacy benchmarks of phase 3 trials: 74.3% (142/191) of participants persisted at 48 weeks, 87.3% (124/142) of persistent patients reached the 2.4 mg/week target dose, mean weight loss in persistent participants reached 14.6% (SD 7.3%), and weight loss followed a steep dose-response gradient from 3.9% in discontinuers to 15.3% at 2.4 mg/week. Third, among persistent participants, clinical phenotype (full-dose attainment, absence of treated OSA, and lower historical lowest reported adult weight) and the IES-2 (the only behavioral predictor surviving FDR correction) were independently associated with the magnitude of weight loss.

### Interpretation and Comparison With Prior Work

Our 48-week persistence rate of 74.3% and full-dose attainment rate of 87.3% substantially exceed figures from large administrative datasets, where 1-year persistence is typically 32% to 50% and full-dose attainment is ≤15% [6-10]. Features of our care setting likely contribute: zero out-of-pocket cost through the early-access program, scheduled clinical visits, a multidisciplinary team, and structured baseline assessment. The dose-response cascade is steep and clinically informative; each step up the titration ladder brings a 3 to 4 percentage-point increment in weight loss, and the 2.4 mg/week plateau yields the full therapeutic effect. Effort directed at supporting persistence and uptitration may therefore yield as much real-world benefit as the molecule’s intrinsic pharmacology.

Translating real-world effectiveness to phase 3 benchmarks requires attention to the population. The step 1 trial enrolled adults with BMI ≥30 kg/m² (mean baseline BMI 37.9 kg/m²) and reported a 14.9% mean weight loss over 68 weeks [4]. Our cohort had a substantially higher baseline BMI (mean 45.7, SD 6.8 kg/m²; all participants ≥40 kg/m²), placing them on average 8 BMI units above the step 1 mean and at the upper end of the obesity-severity spectrum, a population historically more refractory to weight-loss interventions and routinely underrepresented in registration trials. Achieving a comparable 14.6% mean weight loss at 48 weeks among persistent patients, under structured but ambulatory real-world care and with shorter follow-up, is a meaningful demonstration that the pharmacological effectiveness of semaglutide 2.4 mg extends to severe obesity when persistence and full-dose titration are achieved. We do not, however, interpret this comparison as evidence of heterogeneity in biological response because biological-response measures (eg, early GLP-1 pharmacodynamic markers, postprandial satiety response, or change in energy intake) were not collected in this study.

Two nonexclusive interpretations of the engagement-persistence association are equally compatible with our data and cannot be distinguished by mediation analysis or any other purely observational approach (see Multimedia Appendix 3 for the underlying structural reason). First, completion of the requested preclinical assessment may be a passive marker of patient characteristics, including motivation, prioritization, time availability, digital and health literacy, language proficiency, or trust in the care process, which would also drive treatment persistence regardless of the platform. Second, completion may actively contribute to persistence: by structuring patient expectations, by exposing patients to optional educational content (which we could not quantify, as identifiable platform-use logs were not retained), or by giving clinicians a richer baseline picture supporting more personalized early follow-up. Both interpretations produce the same statistical pattern, including the observation that approximately 69% of the engagement-weight-loss association is statistically accounted for by 48-week persistence. The clinical implication, fortunately, does not depend on the mechanism: a measurable marker of patients at risk of early discontinuation is clinically actionable, with low-engagement patients (1-y discontinuation: 40.5%) being flaggable at initiation for targeted counseling, closer follow-up, and proactive tolerability management.

Direct comparisons with previously published digital-engagement studies in pharmacological obesity care are limited. The most comparable evidence concerns digital weight-management programs used as adjuncts to lifestyle intervention or telemedicine-delivered GLP-1 prescribing services, both of which conflate engagement (a behavior) with the intervention (the content delivered). To our knowledge, ours is the first study in adults with severe obesity treated with semaglutide 2.4 mg to operationalize engagement as a preclinical patient behavior measured independently of educational exposure, and the first to report a graded engagement-persistence association in this population. Our results are qualitatively consistent with the recent joint advisory by US obesity societies calling for digital tools as enablers of adherence and equity in GLP-1 therapy [11].

Among persistent patients, full-dose attainment was the dominant positive predictor of weight loss, consistent with the dose response established in pivotal trials [4,5]. Treated OSA predicted smaller weight loss despite continuous positive airway pressure therapy, in agreement with prior studies [26,27], and likely reflecting residual sleep fragmentation or neuroendocrine changes that attenuate metabolic response. A family history of T2D showed a consistent negative signal, plausibly reflecting inherited insulin resistance, altered incretin sensitivity, or ectopic lipid storage that reduces GLP-1 pathway response [28,29]; current T2D status was not itself associated with smaller weight loss in our cohort, possibly because our participants with diabetes were enrolled through an obesity-focused program with a mean baseline HbA_1c_ of 5.9%, representing a phenotype of well-controlled, mildly diabetic severe obesity rather than classical T2D. A higher historically reported lowest adult weight was associated with smaller relative weight loss, consistent with set-point biology [30]: the biologically defended weight may constrain pharmacological response.

In the behavioral subgroup with FDR correction, the IES-2 was the only behavioral predictor approaching the multiplicity-corrected significance threshold, extending prior literature linking intuitive eating to better self-regulation and lower body weight in both general and postbariatric populations [31,32]. To our knowledge, this is the first study to show an independent association between baseline intuitive eating and weight-loss magnitude in adults with severe obesity (BMI ≥40 kg/m²) treated with semaglutide 2.4 mg under structured care. The remaining 5 scales (Binge Eating Scale, Hospital Anxiety and Depression Scale, Perceived Stress Scale, Insomnia Severity Index, and Ricci-Gagnon) were uninformative after multiplicity control, arguing against the use of generic psychological batteries as predictors of semaglutide response in severe obesity and instead supporting a more targeted assessment focused on eating-behavior traits.

### Limitations

Several limitations must be acknowledged. The observational single-center design precludes causal inference, and residual confounding of the engagement-persistence association is likely substantial: completion behavior plausibly covaries with unmeasured factors such as health literacy, digital literacy, French-language proficiency, time and competing-demands availability, broader socioeconomic stability beyond what the 3-level CSP captures, and undiagnosed mental health symptoms, any of which could influence both completion and persistence through pathways unrelated to the platform itself. The mediation analysis is subject to the further limitation that, with observational data, it cannot distinguish a passive-marker interpretation (in which engagement marks patient characteristics that also drive persistence) from an active-aid interpretation (in which engagement contributes to persistence), because both produce the same statistical pattern; the indirect-effect estimate should therefore be read as a descriptive partition of the observed association, not as evidence for either interpretation. The behavioral subgroup (n=91) was self-selected and restricted to participants with complete baseline scales; only the IES-2 association survived FDR correction, and the remaining behavioral results should be interpreted as hypothesis-generating. Gastrointestinal adverse events, the leading documented reason for discontinuation, were not prospectively graded. Engagement was measured only at baseline; longitudinal platform use and individual exposure to optional educational content were not captured because identifiable platform logs were not retained during the study. Missing 48-week weight data (49/191, 25.7%) were concentrated among patients who discontinued; substantive conclusions were unchanged under multiple imputation (Table 2). The “Other” CSP groups retirees, students, homemakers, and unemployed individuals; coding retirees separately did not materially alter the engagement-persistence association (Table S1 in Multimedia Appendix 1). Generalizability is constrained by the single-center French setting, predominantly female participants, high prevalence of treated OSA, and zero-cost access through the early-access program. The 11% prevalence of T2D limits stratified inference in participants with diabetes, although point estimates of the primary predictors were stable across this stratum.

### Conclusions

In adults with severe obesity receiving once-weekly semaglutide 2.4 mg under structured early-access care, real-world effectiveness approached phase 3 benchmarks, with persistence and full-dose attainment identified as the rate-limiting steps. Baseline engagement with a structured digital assessment platform predicted 48-week persistence in a graded manner. Whether the platform passively reflects patient characteristics or actively contributes to persistence cannot be determined from observational data; in either case, engagement is best understood as a clinically useful marker of patients at risk of early discontinuation, observable at the point of treatment initiation. Beyond individual care, these findings support a stratified real-world care model in which patients at highest risk of early discontinuation are identified at initiation through their interaction with structured digital assessment and supported through targeted strategies, including intensified counseling, closer follow-up, and proactive tolerability management, that support persistence and full-dose titration. Multicenter prospective evaluation in more diverse settings, including the cost-effectiveness of stratified-care pathways anchored in baseline engagement, is the natural next step.

## Supplementary material

10.2196/80119 Multimedia Appendix 1 Supplementary tables and figure.

10.2196/80119 Multimedia Appendix 2 Statistical analysis plan.

10.2196/80119 Multimedia Appendix 3 Directed acyclic graph (DAG).

10.2196/80119 Checklist 1 STROBE checklist.

## Abbreviations

CKD-EPI: Chronic Kidney Disease Epidemiology Collaboration
CSP: socioprofessional category
ESS: Epworth Sleepiness Scale
FDR: false discovery rate
FDR: false-discovery-rate
GLP-1 RA: glucagon-like peptide-1 receptor agonist
HbA_1c_: glycated hemoglobin
IES-2: Intuitive Eating Scale-2
OR: odds ratio
OSA: obstructive sleep apnea
OSA: obstructive sleep apnea
STROBE: Strengthening the Reporting of Observational Studies in Epidemiology
T2D: type 2 diabetes
