# Supplementary material for: Digital Engagement and Predictors of Semaglutide Persistence and Weight Loss in Severe Obesity: 48-Week Observational Study
Source: J Med Internet Res. 2026 Aug 26;28:e80119. doi: 10.2196/80119 (PMC13515347; doi:10.2196/80119)
Supplement: Supplementary file 2 [file jmir-v28-e80119-s001.docx]

**Multimedia Appendix 1**

*Supplementary tables*

## **Table S1. Engagement-persistence association under a four-category socio-professional classification with retirees coded separately**

This post-hoc sensitivity analysis was added at the revision stage to assess the robustness of the engagement-persistence association to the heterogeneity of the "Other" socio-professional category used in the primary analysis, which grouped retirees, students, homemakers, and unemployed individuals. Retirees (defined as participants in the "Other" category aged ≥65 years) were re-coded as a separate fourth category, and the primary multivariable logistic regression of 48-week discontinuation on the ordinal engagement variable was re-fitted, adjusted for age, sex, baseline body mass index, socio-professional category, and historical lowest reported adult weight.

| **Specification** | **Adjusted OR per ordinal engagement level** | **95% CI** | **P** |
| --- | --- | --- | --- |
| Primary analysis: 3-category socio-professional (executives / self-employed; employees; other) | 0.60 | 0.39–0.91 | .016 |
| Sensitivity: 4-category socio-professional (executives / self-employed; employees; retirees; other) | 0.59 | 0.39–0.91 | .016 |

*Models fitted with persistence as the outcome and the protective odds ratio (1/exp(β)) reported here for consistency with the main manuscript. Retirees represented 10/72 (13.9%) of the original "Other" category. The direction, magnitude, statistical significance, and 95% confidence interval of the engagement effect were unchanged after separating retirees into their own category, supporting the robustness of the primary finding to this coding decision. OR: odds ratio.*

## **Table S2. Sensitivity of the behavioural-scale univariate associations to inclusion of partial responders, with item-level multiple imputation**

This post-hoc sensitivity analysis was added at the revision stage to assess the robustness of the behavioural-subgroup univariate associations to the restriction, in the primary analysis, to participants with all seven validated baseline scales fully completed (n=91). In this sensitivity analysis, partial responders contributed all available scales; per-scale univariate associations between scale total and 48-week percent weight change were therefore computed on each scale's available-case sample. For partial responders within a given scale, missing items were imputed by item-level multiple imputation (m=20, predictive mean matching within scale; pooling by Rubin's rules); scale totals were then recomputed from the imputed item-level data. Where item-level data could not be reconstructed from the database, the available-case (no item-level imputation) result is reported.

| **Scale** | **n** | **β (% wt change per point), primary (n=91)** | **β, available case (item-level MI)** | **Raw P** | **BH-FDR q** |
| --- | --- | --- | --- | --- | --- |
| Intuitive Eating Scale-2 | 101 | +2.89 | +2.12 | .032 | .089 |
| Epworth Sleepiness Scale | 109 | +0.32 | +0.23 | .071 | .124 |
| HADS total | 97 | −0.15 | −0.17 | .069 | .124 |
| Insomnia Severity Index | 109 | +0.17 | +0.15 | .131 | .184 |
| Perceived Stress Scale | 101 | −0.08 | −0.09 | .293 | .342 |
| Binge Eating Scale | 106 | −0.00 | −0.02 | .804 | .804 |
| Ricci-Gagnon Physical Activity | 98 | −0.00 | +0.01 | .954 | .954 |

*Available-case sample sizes are larger than the n=91 complete-case sample, reflecting the inclusion of partial responders for each scale. Effect-size point estimates were attenuated for the Intuitive Eating Scale-2 (β +2.12 vs +2.89) and the Epworth Sleepiness Scale (β +0.23 vs +0.32) in the relaxed sample. Under Benjamini-Hochberg false-discovery-rate correction across the seven scales, no behavioural scale survived the q < 0.05 threshold in this sensitivity analysis; the IES-2 association approached but did not cross the threshold (q = 0.089). The qualitative finding that IES-2 is the strongest behavioural predictor of weight loss was preserved. BH-FDR: Benjamini-Hochberg false-discovery rate; HADS: Hospital Anxiety and Depression Scale; MI: multiple imputation.*

## **Table S3. Baseline characteristics of the behavioural subgroup (n=91)**

Baseline demographic, anthropometric, clinical, and behavioural characteristics of the pre-specified behavioural subgroup comprising the 91 persistent participants who completed all seven validated baseline behavioural scales.

| **Characteristic** | **Value (n=91)** |
| --- | --- |
| ***Demographics*** | |
| Age (years), mean (SD) | 52.0 (12.2) |
| Male, n (%) | 26 (28.6) |
| ***Anthropometrics*** | |
| Body weight (kg), mean (SD) | 126.1 (24.6) |
| Body mass index (kg/m²), mean (SD) | 45.5 (6.8) |
| Waist circumference (cm), mean (SD) | 130.2 (13.1) |
| Lowest reported adult weight (kg), mean (SD) | 74.9 (19.4) |
| ***Clinical characteristics*** | |
| Hypertension, n (%) | 53 (58.2) |
| Dyslipidaemia, n (%) | 32 (35.2) |
| Treated obstructive sleep apnoea, n (%) | 45 (49.5) |
| Type 2 diabetes, n (%) | 9 (9.9) |
| Family history of type 2 diabetes, n (%) | 27 (29.7) |
| Family history of obesity, n (%) | 53 (58.2) |
| Prior bariatric surgery, n (%) | 22 (24.2) |
| ***Baseline behavioural scales, mean (SD)*** | |
| Intuitive Eating Scale-2 total | 3.55 (0.67) |
| Binge Eating Scale | 14.7 (9.3) |
| Hospital Anxiety and Depression Scale total | 12.8 (7.7) |
| Perceived Stress Scale | 29.2 (8.7) |
| Insomnia Severity Index | 11.4 (6.7) |
| Epworth Sleepiness Scale | 8.3 (5.0) |
| Ricci-Gagnon Physical Activity | 15.6 (8.3) |
| ***48-week outcome*** | |
| Percent weight loss at 48 weeks, mean (SD) | 14.6 (6.9) |

*Data are mean (SD) or n (%). SD: standard deviation.*

## **Table S4. Univariate associations between baseline behavioural scales and 48-week percent weight change, with Benjamini-Hochberg false-discovery-rate correction (n=91)**

| **Scale** | **n** | **β (% wt change per point)** | **SE** | **Raw P** | **Benjamini-Hochberg q** |
| --- | --- | --- | --- | --- | --- |
| Intuitive Eating Scale-2 | 91 | +2.89 | 1.05 | .007 | .051 |
| Epworth Sleepiness Scale | 91 | +0.32 | 0.14 | .025 | .087 |
| Hospital Anxiety and Depression Scale total | 91 | −0.15 | 0.09 | .109 | .223 |
| Insomnia Severity Index | 91 | +0.17 | 0.11 | .128 | .223 |
| Perceived Stress Scale | 89 | −0.08 | 0.09 | .369 | .517 |
| Binge Eating Scale | 86 | −0.00 | 0.08 | .979 | .983 |
| Ricci-Gagnon Physical Activity | 91 | −0.00 | 0.09 | .983 | .983 |

*β: regression coefficient (percentage-point change in 48-week percent weight loss per one-point increment in scale total); SE: standard error; raw P: uncorrected two-sided P-value; q: false-discovery rate-corrected significance threshold (Benjamini and Hochberg). q < 0.05 indicates the association surviving multiplicity correction.*

## **Table S5. Multivariable linear regression of 48-week percent weight change in the behavioural subgroup (n=91), adjusted for clinical confounders**

| **Predictor** | **β** | **SE** | **95% CI** | **P** |
| --- | --- | --- | --- | --- |
| Intuitive Eating Scale-2 (per point) | +2.27 | 0.97 | +0.34 to +4.19 | .022 |
| Epworth Sleepiness Scale (per point) | +0.33 | 0.12 | +0.08 to +0.58 | .009 |
| Treated obstructive sleep apnoea (yes vs no) | −4.37 | 1.25 | −6.85 to −1.88 | .001 |
| Family history of type 2 diabetes (yes vs no) | −3.34 | 1.37 | −6.07 to −0.61 | .017 |
| Historical lowest adult weight (per kg) | −0.09 | 0.04 | −0.17 to −0.01 | .023 |
| Age (per year) | −0.04 | 0.06 | −0.15 to +0.07 | .486 |
| Male sex | −1.17 | 1.68 | −4.51 to +2.17 | .487 |
| Baseline body mass index (per kg/m²) | +0.03 | 0.10 | −0.17 to +0.22 | .802 |

*Outcome: percent weight change at 48 weeks (positive values denote greater weight loss). Adjusted R² = 0.27; F-test P < .001. The model includes the two behavioural predictors with raw P < .10 in univariate testing (Intuitive Eating Scale-2 and Epworth Sleepiness Scale) together with the a priori clinical confounders treated obstructive sleep apnoea, family history of type 2 diabetes, historical lowest reported adult weight, age, sex, and baseline body mass index. β: regression coefficient; SE: standard error.*

## **Table S6. Sensitivity analysis stratified by baseline type 2 diabetes status**

| **Outcome** | **T2D at baseline (n=21)** | **No T2D at baseline (n=170)** | **P** |
| --- | --- | --- | --- |
| 48-week persistence, % | 76.2 | 74.1 | .83 |
| Full-dose attainment, n/N (%) among persistent | 15/16 (93.8) | 109/126 (86.5) | .69 |
| Percent weight loss at 48 weeks, mean (SD) | 8.9 (7.4) | 12.2 (8.4) | .07 |
| Reached ≥10% weight loss, % | 38.1 | 58.2 | .10 |
| Reached ≥15% weight loss, % | 19.0 | 37.6 | .11 |

*P from chi-square test for categorical outcomes and Welch t-test for continuous outcomes. The direction and statistical significance of all primary predictor effects, including the engagement-persistence and mediation analyses, were unchanged when stratifying by baseline T2D status. T2D: type 2 diabetes; SD: standard deviation.*

## **References**

1. Benjamini Y, Hochberg Y. Controlling the false discovery rate: a practical and powerful approach to multiple testing. J R Stat Soc Series B Stat Methodol. 1995;57(1):289-300.

2. Rubin DB. Multiple imputation for nonresponse in surveys. New York, NY: John Wiley & Sons; 1987.

3. von Buuren S, Groothuis-Oudshoorn K. mice: multivariate imputation by chained equations in R. J Stat Softw. 2011;45(3):1-67. doi:10.18637/jss.v045.i03
