# Supplementary material for: Digital Engagement and Predictors of Semaglutide Persistence and Weight Loss in Severe Obesity: 48-Week Observational Study
Source: J Med Internet Res. 2026 Aug 26;28:e80119. doi: 10.2196/80119 (PMC13515347; doi:10.2196/80119)
Supplement: Supplementary file 3 [file jmir-v28-e80119-s002.docx]

**Multimedia Appendix 2**

*Statistical Analysis Plan*

*This appendix documents the statistical analysis plan for the present manuscript. It covers the analytic population, exposure definition, outcomes, covariates, statistical methods (including the engagement-persistence model, dose-response analysis, mediation analysis, behavioural subgroup analysis with multiplicity control, and missing-data strategy), analytical roles, and reporting conventions.*

# **1. Study design and analytic population**

Prospective single-centre observational cohort study. The analytic population comprised all adults (≥18 years) with body mass index (BMI) ≥40 kg/m² and at least one obesity-related complication who consecutively initiated semaglutide 2.4 mg/week through the French early-access programme (Accès Précoce) at the Nutrition-Diabetes Department of Montpellier University Hospital between April 2022 and April 2023, after applying the pre-specified exclusion criteria (prior glucagon-like peptide-1 receptor agonist exposure at therapeutic doses, monogenic/syndromic/secondary obesity, pregnancy or planned bariatric surgery during follow-up, active cancer, estimated glomerular filtration rate <30 mL/min/1.73 m²).

Follow-up was conducted over 48 weeks, with scheduled clinical visits at baseline and at weeks 12, 24, and 48. Sampling was exhaustive within the recruitment window.

# **2. Exposure**

The primary exposure of interest was baseline engagement with the Aviitam® digital assessment platform, operationalised as an ordinal three-level variable: (0) the patient had never used the platform; (1) the patient had used the platform but had not completed all seven validated patient-reported scales; (2) the patient had completed all seven validated patient-reported scales. Pathway completion is an autonomous, pre-clinical patient behaviour and was assessed before the first clinical visit.

The ordinal engagement variable was used as the primary exposure for this revision (the original submission used a binary specification, which is retained here as a sensitivity exposure).

# **3. Outcomes**

### ***3.1 Primary outcome***

Treatment persistence at 48 weeks, defined as the patient still receiving semaglutide at any dose at the 48-week visit.

### ***3.2 Secondary outcomes***

(i) Attainment of the 2.4 mg/week target dose among persistent participants. (ii) Percent body weight change at weeks 12, 24, and 48, computed as 100 × (baseline weight − measured weight) / baseline weight. (iii) Dose-response relationship between the semaglutide dose maintained at 48 weeks (≤1 mg, 1.7 mg, 2.4 mg, or discontinued) and percent weight change at 48 weeks. (iv) Clinical, metabolic, and behavioural predictors of percent weight change among persistent participants.

# **4. Covariates**

A priori adjustment covariates: age (continuous), sex (male / female), baseline body mass index (continuous), and socio-professional category (3 levels). Additional covariates entered the multivariable models if associated with the outcome at P ≤ .10 in univariate testing: family history of type 2 diabetes (yes/no), family history of obesity (yes/no), treated obstructive sleep apnoea (yes/no), baseline type 2 diabetes (yes/no), prior bariatric surgery (yes/no), historical lowest reported adult weight (continuous, kg), and baseline glycated haemoglobin (continuous, %).

# **5. Statistical methods**

### ***5.1 Descriptive analyses***

Continuous variables are reported as mean (standard deviation) or, where the distribution is markedly non-normal, median (interquartile range). Categorical variables are reported as n (%). Normality is assessed by Shapiro-Wilk testing. Baseline characteristics are compared across engagement levels using Student t-tests, Wilcoxon rank-sum tests, Cochran-Armitage trend tests, or chi-square tests as appropriate.

### ***5.2 Engagement-persistence association***

Logistic regression of 48-week discontinuation on the ordinal engagement variable (continuous trend predictor), adjusted for age, sex, baseline BMI, socio-professional category, and any further covariate associated with persistence at P ≤ .10 in univariate testing. Adjusted odds ratios and 95% confidence intervals are reported. A binary specification of engagement (full completion vs. not) is reported as a sensitivity analysis. Model discrimination is summarised by the C-statistic.

### ***5.3 Dose-response in weight loss***

Percent weight change at 48 weeks stratified by the semaglutide dose maintained at 48 weeks (discontinued / ≤1 mg / 1.7 mg / 2.4 mg). Trend tested using Cochran-Armitage trend test. Weight-loss trajectories over time are displayed using mean weights at weeks 0, 12, 24, and 48 within each dose category.

### ***5.4 Mediation analysis***

Formal mediation analysis decomposing the effect of baseline engagement on 48-week percent weight change into a direct effect (c') and an indirect effect mediated by 48-week persistence (a × b), following the Baron-Kenny framework. Bootstrap 95% confidence intervals are computed for the indirect effect using 1,000 resamples (Preacher and Hayes). The mediation analysis is fitted with both ordinal and binary engagement exposures, adjusted for age, sex, and baseline BMI.

### ***5.5 Behavioural-subgroup analysis***

Among persistent participants who completed all seven validated baseline behavioural scales (Intuitive Eating Scale-2, Binge Eating Scale, Hospital Anxiety and Depression Scale total, Perceived Stress Scale, Epworth Sleepiness Scale, Insomnia Severity Index, Ricci-Gagnon Physical Activity Questionnaire), the univariate associations between each scale total and 48-week percent weight change are tested by linear regression. Multiple testing across the seven scales is controlled using the Benjamini-Hochberg false-discovery-rate procedure; only associations with q < 0.05 are interpreted as surviving multiplicity correction. A multivariable linear model is fitted including the behavioural predictors with raw P < .10 in univariate testing together with the a priori clinical confounders age, sex, baseline BMI, treated obstructive sleep apnoea, family history of type 2 diabetes, and historical lowest reported adult weight.

### ***5.6 Missing-data strategy***

Missing 48-week weight data are handled by last-observation-carried-forward (LOCF) as the primary approach, consistent with routine practice in the Department. A sensitivity analysis using multiple imputation by chained equations (m = 20) is conducted with the IterativeImputer implementation in scikit-learn (Bayesian Ridge per-variable estimator, sample-from-posterior). Auxiliary variables in the imputation model: age, sex, baseline BMI, baseline weight, ordinal engagement, persistence status, full-dose attainment, treated obstructive sleep apnoea, family history of type 2 diabetes, baseline type 2 diabetes, historical lowest adult weight, baseline HbA1c. Estimates and standard errors are pooled across imputations using Rubin's rules; degrees of freedom for confidence intervals are adjusted using the Rubin-Schenker approximation. A complete-case analysis is reported for transparency. For partial behavioural-scale responders, an item-level multiple-imputation sensitivity analysis is conducted (m = 20, predictive mean matching within scale).

### ***5.7 Subgroup analyses***

Sensitivity analyses stratified by baseline type 2 diabetes status are planned, with the primary engagement-persistence association and mediation analyses re-fitted within each stratum.

### ***5.8 Statistical thresholds***

All pre-specified hypothesis tests use a two-sided α of 0.05. For exploratory behavioural-scale analyses, multiplicity is controlled at q < 0.05 (Benjamini-Hochberg FDR). Confidence intervals are reported at the 95% level.

### ***5.9 Software***

Primary analyses are performed in R version 4.3.3 (R Foundation for Statistical Computing). The Baron-Kenny mediation bootstrap and the multiple-imputation sensitivity analysis are performed in Python 3.10 (scikit-learn, statsmodels, scipy). Random seeds are documented in the analysis scripts.

# **6. Analytical roles**

Primary statistical analyses are conducted by an independent statistician (J. Anitcheou) with no financial interest in the Aviitam® platform. The corresponding author (A. Avignon), who is a shareholder of Aviitam®, did not participate in analytical decisions regarding engagement-related analyses. This analysis plan was finalised before any analytical re-run conducted in the revision of the manuscript; analyses added at the revision stage (notably the ordinal engagement specification, the Baron-Kenny mediation analysis, the multiple-imputation sensitivity analysis for missing 48-week weight outcomes, the item-level imputation sensitivity for partial behavioural responders, the four-category socio-professional sensitivity analysis, and the stratification by baseline type 2 diabetes status) are documented here for reference rather than presented as part of the original pre-submission plan.

# **7. Reporting**

Results are reported following the STROBE statement for observational studies. Exact P-values are reported to three decimal places, except for P < .001 (reported as such) and P ≥ .99 (reported as .99). Effect sizes are reported with point estimates and 95% confidence intervals.
