# Supplementary material for: Digital Engagement and Predictors of Semaglutide Persistence and Weight Loss in Severe Obesity: 48-Week Observational Study
Source: J Med Internet Res. 2026 Aug 26;28:e80119. doi: 10.2196/80119 (PMC13515347; doi:10.2196/80119)
Supplement: Supplementary file 4 [file jmir-v28-e80119-s003.docx]

**Multimedia Appendix 3**

**Directed Acyclic Graph (DAG) of the engagement-persistence-weight loss analyses**

**Figure A3. Directed acyclic graph of the engagement-persistence-weight loss analyses, showing measured adjustment and residual unmeasured confounding.**

The exposure of interest is baseline engagement (E) with the Aviitam® pre-clinical assessment. The mediator (M) is treatment persistence at 48 weeks. The outcome (Y) is percent body weight change at 48 weeks. The a-path (E → M), b-path (M → Y), and direct c'-path (E → Y) constitute the mediation triangle whose numerical estimates are reported in Figure 4 of the main text and in Table 2. Measured baseline characteristics (C) — age, sex, baseline body mass index, socio-professional category, treated obstructive sleep apnoea, family history of type 2 diabetes, and historical lowest reported adult weight — are entered as adjusted confounders in the multivariable models (solid arrows from C to E, M, and Y). Unmeasured patient characteristics that plausibly co-vary with completion behaviour, with treatment persistence, and with weight loss (U: health literacy, digital literacy, French-language proficiency, time and competing-demand availability, broader socioeconomic stability beyond the three-level socio-professional category, and undiagnosed mental health symptoms) are represented by a single unmeasured-confounder cluster (dashed arrows from U to E, M, and Y; not adjustable in the present data). The U → Y pathway captures plausible direct effects of unmeasured patient characteristics on weight loss that bypass persistence — for instance, health literacy or socioeconomic stability influencing dietary self-management independently of whether semaglutide is continued.

**Conventions.**

Solid arrows represent associations adjusted for in the analysis. Dashed arrows represent pathways through unmeasured factors that cannot be adjusted for in the present data. C: measured confounders. U: unmeasured confounders. E: exposure (baseline engagement). M: mediator (48-week persistence). Y: outcome (% weight change at 48 weeks). a, b, c': path coefficients of the mediation analysis.

**Interpretive note.**

The U → E and U → M pathways through unmeasured common causes are the structural reason why the mediation analysis cannot adjudicate between a passive-marker interpretation (in which the observed engagement-persistence association is generated by the common causes U rather than by a direct effect of E on M) and an active-aid interpretation (in which the observed association is driven, at least in part, by a direct E → M causal effect). Both structures produce the same observed statistical pattern in this data. The additional U → Y pathway means that residual confounding of the engagement-weight loss association is plausible even after adjustment for the measured confounders C, which further supports a descriptive rather than causal reading of the c'-path estimate. These limitations are acknowledged explicitly in the main text Limitations section.
